# Preparation of MgGa Layered Double Hydroxides and Possible Compositional Variation

**DOI:** 10.3390/nano11051206

**Published:** 2021-05-01

**Authors:** Rattanawadee (Ploy) Wijitwongwan, Soontaree (Grace) Intasa-ard, Makoto Ogawa

**Affiliations:** 1 School of Energy Science and Engineering, Vidyasirimedhi Institute of Science and Technology (VISTEC), 555 Moo 1 Payupnai, Wangchan, Rayong 21210, Thailand; Rattanawadee.W_s17@vistec.ac.th; 2 School of Molecular Science and Engineering, Vidyasirimedhi Institute of Science and Technology (VISTEC), 555 Moo 1 Payupnai, Wangchan, Rayong 21210, Thailand; soontaree.int@gmail.com

**Keywords:** layered double hydroxides, layer charge density

## Abstract

Layered double hydroxides (LDHs), shown as the general formula of [M^2+^_1−x_M^3+^_x_(OH)_2_]^x+^(A^n−^)_x/n_∙yH_2_O, are useful for various applications such as anion exchangers/adsorbents, catalysts and catalysts’ supports, and drug/gene carriers due to their structural, compositional and morphological characteristics and their variation. The x value (M^3+^/(M^2+^ + M^3+^) ratio) in layered double hydroxides (LDHs), corresponding to the layer charge density, is one of the important parameters for controlling the properties of LDHs. The x values in commonly available LDHs are limited (0.2 < x < 0.3). In order to obtain LDHs with x < 0.2, Mg^2+^ Ga^3+^–LDHs with interlayer iodide were examined. The linear correlation between lattice parameter *a* and x value in the products with x of 0.06–0.24 was seen, suggesting the successful substitution of Mg^2+^ in the brucite-like sheet with Ga^3+^. Carbonate and dodecyl sulfate types MgGa–LDH were prepared by ion exchange with carbonate anion and reconstruction in aqueous solution of sodium dodecyl sulfate. The products with x of 0.06 were dispersed in water and hexanol better than those with x of 0.24 for MgGa–LDHs containing carbonate and dodecyl sulfate, respectively, suggesting effects of the lower layer charge density on the dispersion.

## 1. Introduction

Layered double hydroxides (LDHs) are a class of layered materials consisting of positively charged brucite-like sheets of metal hydroxide, where some of divalent metal cations (M^2+^) are substituted with trivalent metal cations (M^3+^) to give positive charge as [M^2+^_1−x_M^3+^_x_(OH)_2_]^x+^, and the charge compensating interlayer exchangeable anions (A^n−^). The overall composition is expressed as the following general formula of [M^2+^_1−x_M^3+^_x_(OH)_2_]^x+^(A^n−^)_x/n_∙yH_2_O. Owing to the versatile compositional variation of LDHs by the selection of metal ions in the hydroxide sheets and the interlayer anions, as well as their quantity (x values in [M^2+^_1−x_M^3+^_x_(OH)_2_]^x+^(A^n−^)_x/n_), possible applications of LDHs have been reported such as polymer additives [1], drug/gene carriers [2,3], catalysts and catalysts’ supports [4,5] and anion exchangers/adsorbents [6,7,8]. Various synthetic methods have been examined and developed to prepare LDHs with desired composition and morphology [9,10].

One of the important parameters for controlling the properties of LDHs is the x value, which corresponds to the anion exchange capacity as well as the layer charge density. Preparation of LDHs with varied x values has been examined and the values of 0.2 < x < 0.3 are commonly approved for pure LDH phases. Apart from the range of 0.20–0.33, products are normally mixtures of LDHs and impurity phases [11,12]. These mixtures were obtained in the preparation of MgAl–LDHs [13,14,15,16,17,18,19], CaAl–LDHs [20], CaFe–LDHs [21], CoFe–LDHs [22,23], and NiAl–LDHs [24] with x < 0.20. It was thought that the limitation of the x value in pure LDHs was caused by a difference in the ionic radii of divalent and trivalent metal cations in framework of LDHs and a large distance between the adjacent interlayer anion in the interlayer space [12].

In order to obtain an LDH with x < 0.20, M^2+^ and M^3+^ shall have similar ionic radii to obtain the substitution of M^2+^ in the brucite-like sheet with M^3+^ without phase separation [11]. If compared with CaAl–LDHs, CaFe–LDHs, MgAl–LDHs, and NiAl–LDHs, which have been extensively investigated among available LDHs, MgGa–LDH is composed of similar size ions ((R_M_^2+^ − R_M_^3+^)/R_M_^2+^ = 47% for CaAl, 36% for CaFe, 26% for MgAl, 22% for NiAl, and 13% for MgGa) [25], while the preparation of MgGa–LDHs has been scarcely reported [26,27,28]. Besides the combination of M^2+^ and M^3+^ in the framework of LDHs, a large distance between the adjacent interlayer anions for LDHs with smaller x values may cause a collapse of the interlamellar domain, leading to the phase segregation [11]. Sasaki and his coworkers have proposed the template concept of interlayer cation to obtain lepidocrocite-type layered titanates (A_y_Ti_2−y/3_Li_y/3_O_4_) with varied layer charge densities. The lepidocrocite-type layered titanates are one group of layered materials consisting of negatively charged titanate sheets and the charge-balancing interlayer cations. It has been reported that the lepidocrocite-type layered titanates with varied y values were obtained using different interlayer cations (A = Cs, Rb and K) and larger cation gave smaller y values [29].

In this study, iodide (0.21 nm) was used as the interlayer anion to avoid possible collapse of the layered structures of MgGa–LDHs due to the size of iodide. Once layered structures of the MgGa–LDHs with varied x formed by coprecipitation, carbonate and dodecyl sulfate forms of the MgGa–LDHs were prepared by the anion exchange with carbonate anion and the reconstruction in aqueous solution of sodium dodecyl sulfate after calcination. The effect of x value on the dispersions of the MgGa–LDHs in water for the carbonate form and in hexanol for the dodecyl sulfate form was examined by the observation of the sedimentation of the solids before and after the storage without agitation.

## 2. Materials and Methods

### 2.1. Materials

Magnesium nitrate hexahydrate (Mg(NO_3_)_2_ 6H_2_O) was obtained from Sigma-Aldrich. Gallium nitrate hydrate (Ga(NO_3_)_3_ xH_2_O, 99.9%) was purchased from Alfa Aesar. Potassium iodide (KI) and sodium carbonate (Na_2_CO_3_) were supplied by Merck Millipore. Potassium hydroxide (KOH) was obtained from Carlo Erba Reagents. Brucite (Mg(OH)_2_) was obtained from Nacalai Tesque. Sodium dodecyl sulfate (C_12_H_25_NaO_4_S, >95.0%), hexanol (C_6_H_14_O, >98.0%), and benzene (C_6_H_6_, >99.5%) were supplied by Tokyo Chemical Industry Co., Ltd. These chemicals were used without further purification. Deionized water was obtained from a Milli-Q system (18.2 MΩ-cm, Merck Millipore, Darmstadt, Germany).

### 2.2. Synthesis of MgGa–LDHs Intercalated with Iodide

**Scheme 1:**
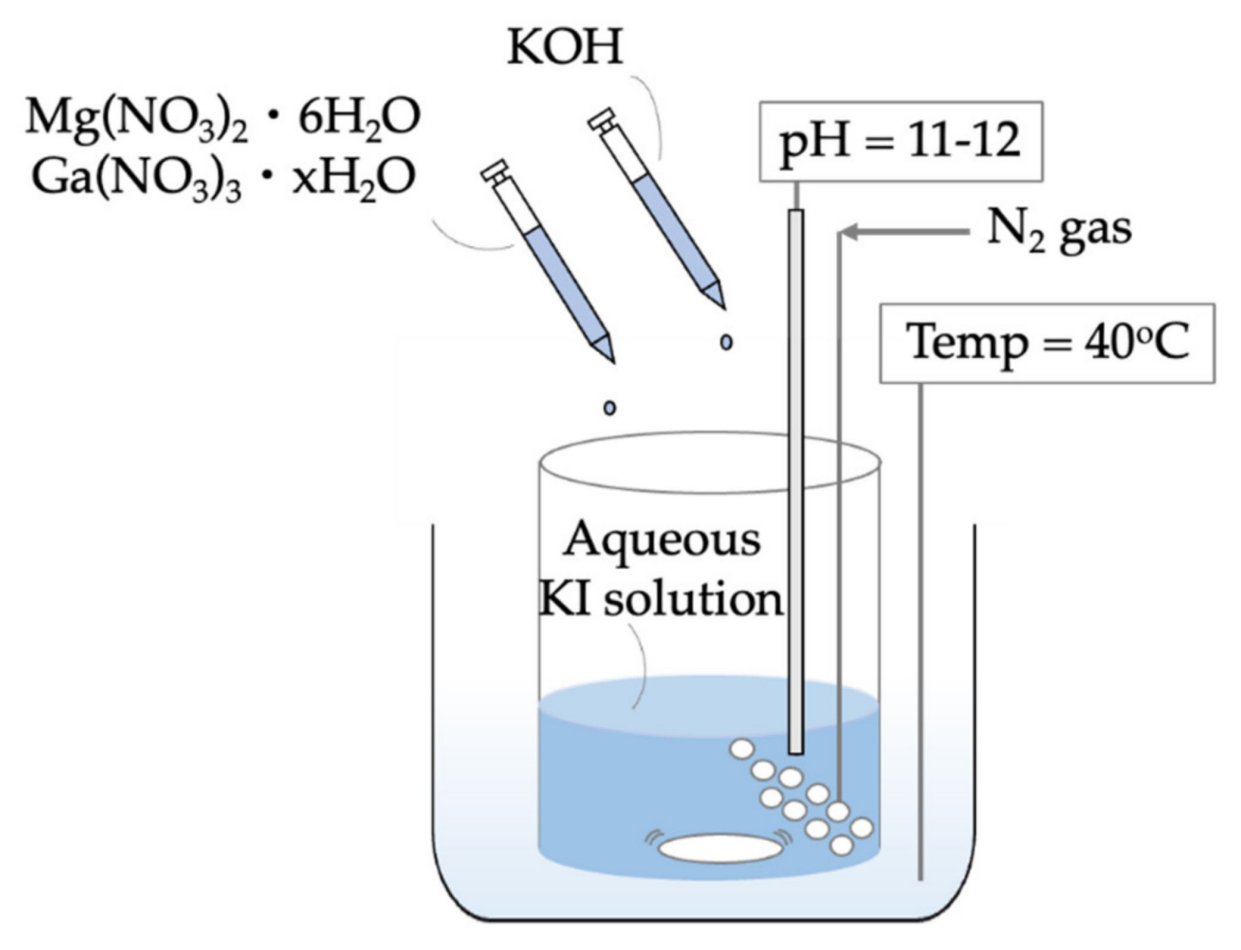
Experimental setup for the synthesis of MgGa–LDHs intercalated with iodide.

MgGa–LDHs with varied x were prepared by coprecipitation from aqueous solutions at controlled pH. The starting solution was prepared by dissolving 3.84 g of Mg(NO_3_)_2_ 6H_2_O and 1.28 g of Ga(NO_3_)_3_ xH_2_O in 20 mL of deionized water for the preparation of the MgGa–LDH with x = 0.25. The starting solution was added dropwise to 40 mL of aqueous solution containing KI (the mole of KI is two times of the total nitrate from the metal salts) with vigorous stirring at 40 °C under nitrogen gas bubbling. The pH of the mixture was kept at 11–12 through the reaction by addition of 0.9 M aqueous solution of KOH as schematically shown in Scheme 1. The MgGa–LDHs were prepared using the same procedure by varying the x of 0.20, 0.13, 0.10, 0.09, and 0.08 in the starting solution. After the reaction, the precipitate was separated from the suspension by centrifugation at 10,000 rpm for 10 min and washed several times with deionized water. Half of the precipitate was dried at 80 °C for 24 h in oven and another half was freeze dried at −108 °C for 24 h. The products thus obtained by the coprecipitation followed by drying in oven and in freeze-dryer are designated as MgGa(I)-x and MgGa(I)-x_F, respectively, where x is the ratio of Ga^3+^/(Mg^2+^ + Ga^3+^) in the product as determined by ICP analysis.

### 2.3. Anion Exchange from Iodide to Carbonate in MgGa–LDHs

To the aqueous solution of Na_2_CO_3_ (1.27 g in 60 mL) 0.3 g of MgGa(I)-x_F (x = 0.24 and 0.06) was added and shaken for 3 h at room temperature. The solid was separated by the centrifugation at 10,000 rpm for 10 min, washed several times with deionized water, and freeze dried. The products are designated as MgGa(CO_3_)-x_F.

### 2.4. Reconstruction of MgGa–LDHs in Aqueous Solution of Sodium Dodecyl Sulfate

MgGa(CO_3_)-x_F (x = 0.24 and 0.06) (0.05 g) were calcined at 500 °C in air under ambient pressure (the heating rate of 10 °C/min), and then, allowed to react with the aqueous solution of sodium dodecyl sulfate (0.50 and 0.15 g of sodium dodecyl sulfate in 25 mL of deionized water for the samples with x of 0.24 and 0.06, respectively), then shaken for 10 h at room temperature. After the centrifugation at 10,000 rpm for 10 min, the solid was washed several times with deionized water, and freeze dried. The products are designated as MgGa(DS)-x_F.

### 2.5. Preparation of Suspension

In total, 10 mg of sample was added to 10 mL of deionized water or hexanol in a vial and sonicated (using Sonorex Digitec, Bandelin) for 2 min.

### 2.6. Characterization

Powder X-ray diffraction (XRD) patterns of the products were recorded at the scan speed of 5.47°/min with step size of 0.1002° using New D8 Advance instrument (Bruker, Billerica, Massachusetts, United States) equipped with Ni filtered Cu Kα radiation (λ = 0.15406 nm). Infrared spectra were obtained using Frontier Fourier transform infrared spectrophotometer (PerkinElmer, Inc., Waltham, MA, USA) by attenuated total reflection (ATR) mode with the resolution of 4.0 cm^−1^. In total, 32 scans were recorded for each sample. The composition was determined by ICP analysis (700 Series ICP-OES, Agilent Technologies, Santa Clara, CA, USA) after dissolving a weighed amount of sample with 1 M aqueous solution of HCl. The zeta potential was measured 3 times at pH of 10–10.5 without any additives using NanoPlus instrument (Micromeritics, Norcross, GA, USA). Prior to the measurement, 3.5 mg of sample was dispersed in 10 mL of deionized water under sonication for 30 min. Scanning electron micrographs (SEM) were obtained on a JSM-7610F field emission scanning electron microscope (JEOL, Ltd., Tokyo, Japan) without a coating.

## 3. Results and Discussion

**Figure 1:**
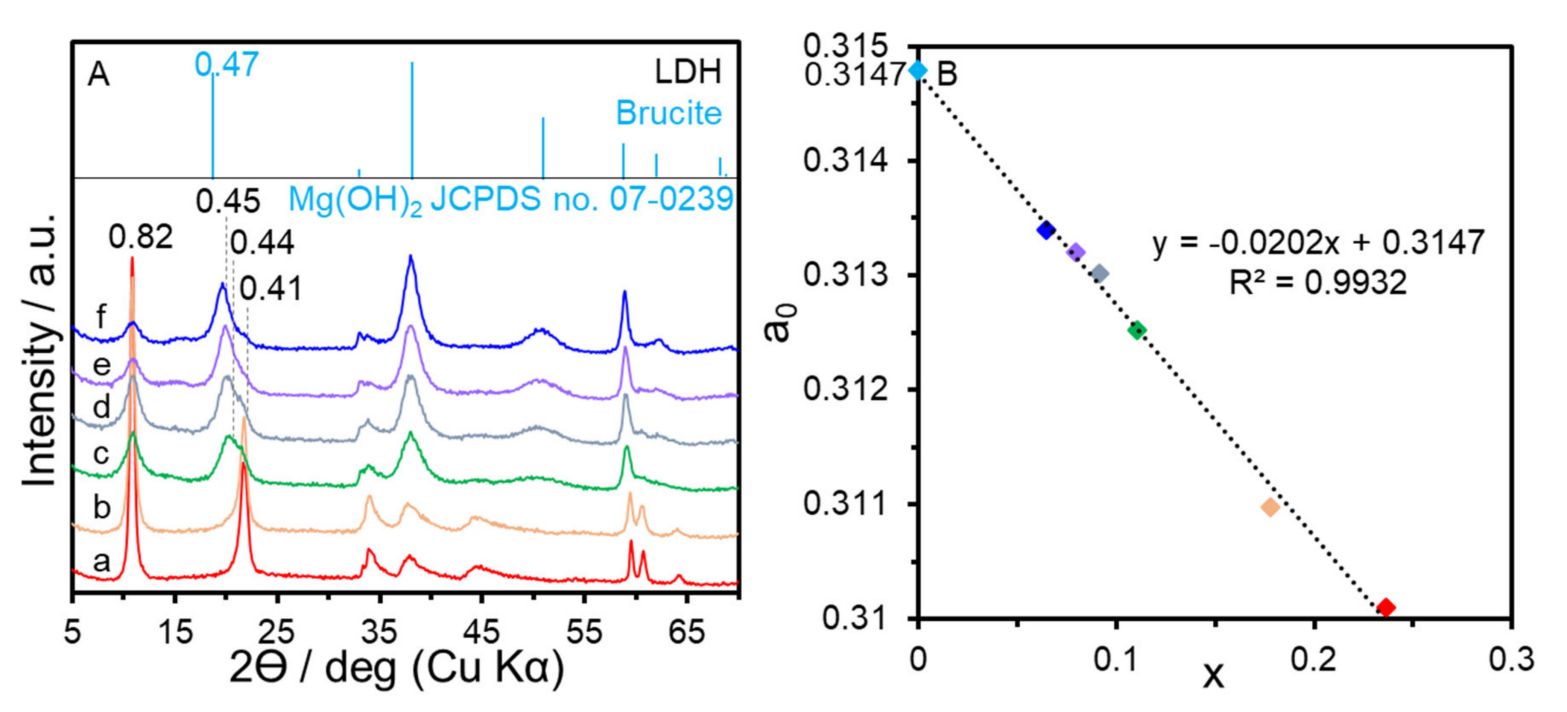
(**A**) XRD patterns of MgGa(I)-x (x = 0.24 (a), 0.18 (b), 0.11 (c), 0.10 (d), 0.08 (e), and 0.06 (f)). (**B**) Relation between the lattice parameter *a* and the x value in the MgGa–LDHs.

The XRD patterns of MgGa(I)-x (x = 0.24, 0.18, 0.11, 0.10, 0.08, and 0.06) are shown in Figure 1A. For MgGa(I)-x (x = 0.24, 0.18), reflections characteristic to the hydrotalcite structure were seen and the segregation of brucite, which has been seen in the previous reports on the preparation of MgAl–LDHs [13,14,15,16,17,18,19], CoFe–LDHs [22,23], and NiAl–LDHs [24] with the x < 0.2, was not observed. The reflections with the d values of 0.82 and 0.41 nm were seen, which are consistent with the d*(003)* and d*(006)* spacings of the reported MgAl–LDHs containing iodide as the interlayer anion, respectively [30].

**Figure 2:**
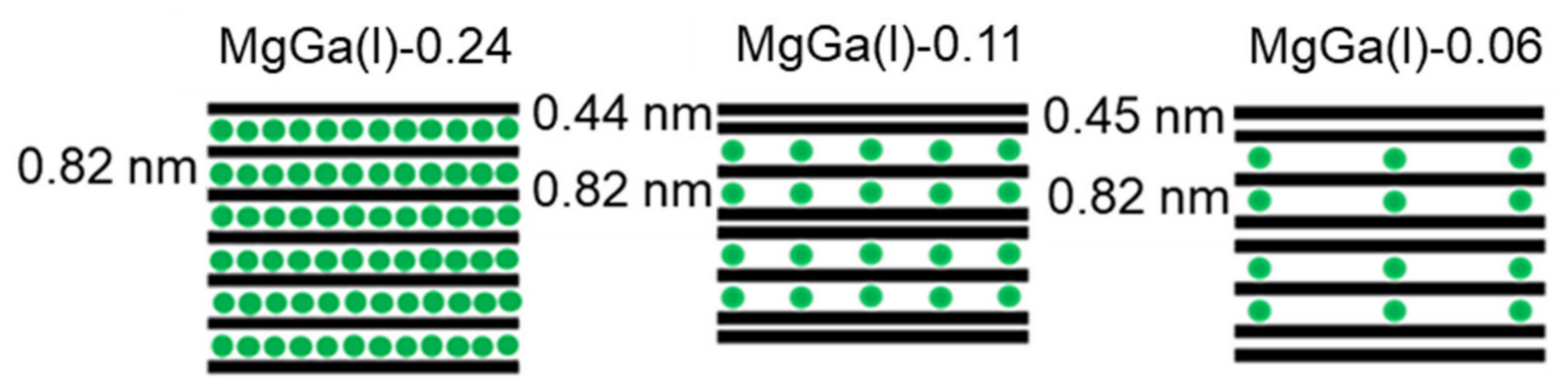
Proposed structure images of MgGa(I)-x (x = 0.24, 0.11, and 0.06).

For the products with smaller x values (x = 0.11, 0.10, 0.08, and 0.06), the basal reflection of brucite was not observed. A reflection at 2*θ* of 20° and characteristic reflections of brucite at 2*θ* of 38, 50, 62° (JCPDS No. 07-0239) were seen together with the characteristic reflections of the hydrotalcite with the basal spacing of 0.82 nm (0.82/0.41 nm series) in Figure 1A(c–f). The difference in the XRD patterns suggested that the x value in MgGa(I)-x, where x value corresponds to the quantity of interlayer anion, affected the crystal structure of the products. The irregular stacking sequence of LDHs has been proposed on CoFe–LDH with 0.14 < x < 0.2 prepared by the post-synthetic oxidation [23]. A reflection revealing the occurrence of the interstratified structure was not observed as proposed by Sasaki [23], it was thought to be caused from the difference in the synthetic methods and the conditions, which affect crystallinity of the products. These observations suggested that MgGa(I)-x (x = 0.11, 0.10, 0.08, and 0.06) had an interstratified structure as proposed in Figure 2.

The lattice parameter *a*, which corresponds to the metal–metal distance in the hexagonal framework of brucite-like sheet [12], can be used to determine the composition from the correlation between the lattice parameter *a* and x value. The lattice parameter was smaller when the x values in the products were larger because of the substitution of larger Mg^2+^ ion (0.072 nm) by smaller Ga^3+^ ion (0.062 nm), where the radii of Mg^2+^ and Ga^3+^ with coordination number of 6 were reported by Shannon [25]. The correlation between *a* and x value is shown in Figure 1B, where linear correlation is seen, suggesting the successful substitution of Mg in the brucite-like sheet with Ga. The correlation was extrapolated to x = 0 to obtain *a* value of 0.3147 nm, which is the same as the lattice parameter *a* of brucite (0.3147 nm, JCPDS No. 07-0239). In the previous studies on MgAl–LDHs, NiAl–LDHs and NiFe–LDHs with x values of 0.1–0.3, the lattice parameter *a* extrapolated at x = 0 gave smaller values (0.3108 [14] and 0.3125 nm [18] for MgAl–LDHs and 0.3093 nm [31] for NiAl– and NiFe–LDHs) than those of Mg(OH)_2_ and Ni(OH)_2_, suggesting that some divalent cations in the brucite-like layer were not substituted with trivalent cations and precipitated separately as metal hydroxides. For the reported carbonate type MgGa–LDHs [32,33] and iodide type CoFe–LDHs [22,23] with x < 0.2, the products were mixtures of LDHs and brucite. In the present study, the MgGa–LDHs (x = 0.06–0.24) containing iodide were obtained without segregation of brucite, which were thought to be possible by the matching of ionic radii of Mg^2+^ and Ga^3+^ as well as by the template effect of iodide, which prevented the collapse of LDH.

**Figure 3:**
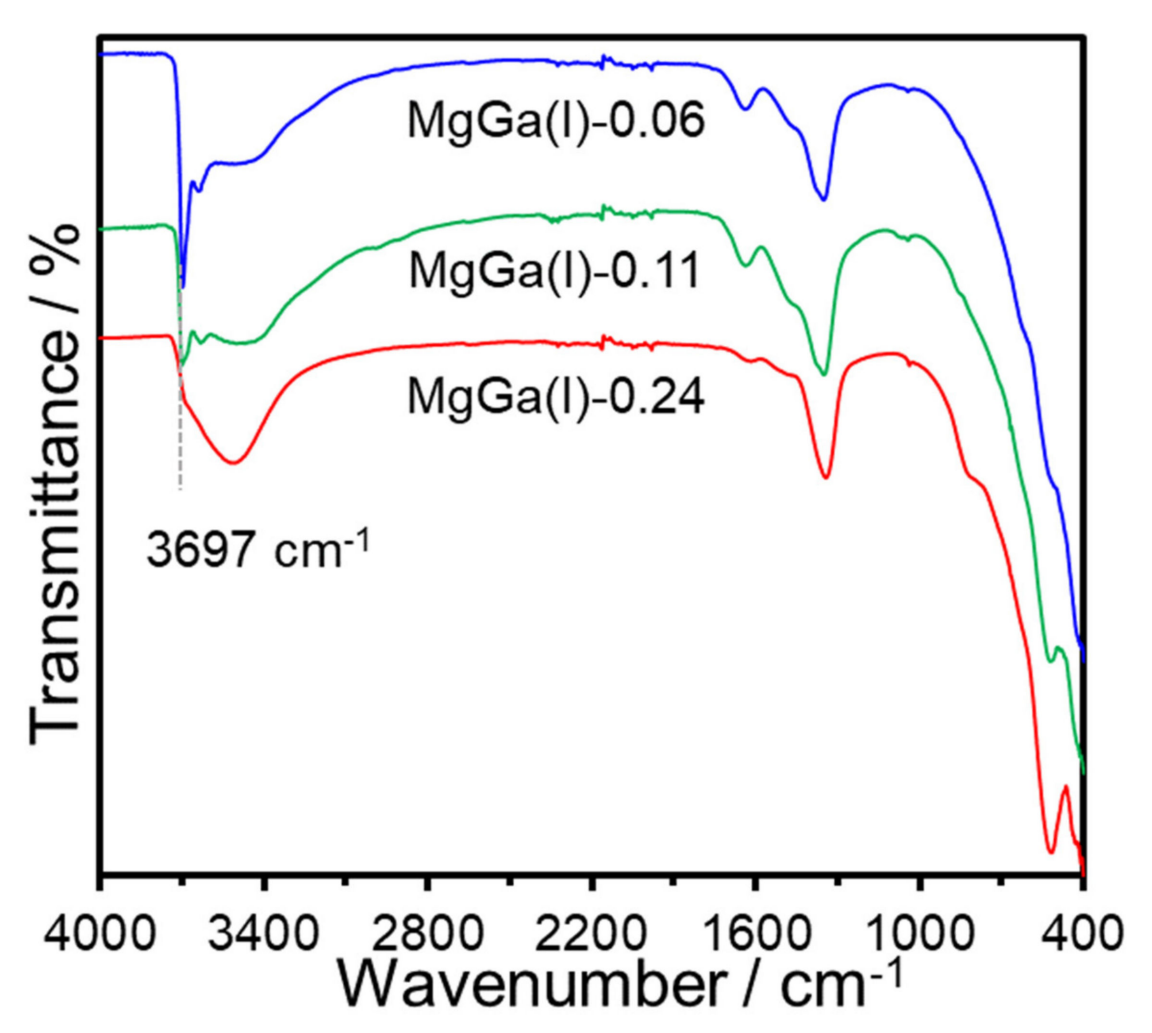
IR spectra of MgGa(I)-x (x = 0.24, 0.11, and 0.06).

Figure 3 shows the IR spectra of MgGa(I)-x (x = 0.24, 0.11, and 0.06). The absorption at around 3400–3600 cm^−1^ was attributed to the OH stretching vibration of hydroxyl groups hydrogen bonded with water molecules and interlayer anion. The absorption at 3697 cm^−1^, which was assigned to free hydroxyl groups in the brucite-like sheet [34], was seen for MgGa(I)-0.11 and MgGa(I)-0.06. These IR results supported the proposed stacking sequence of the products (x = 0.24, 0.11, and 0.06) (Figure 2).

**Figure 4:**
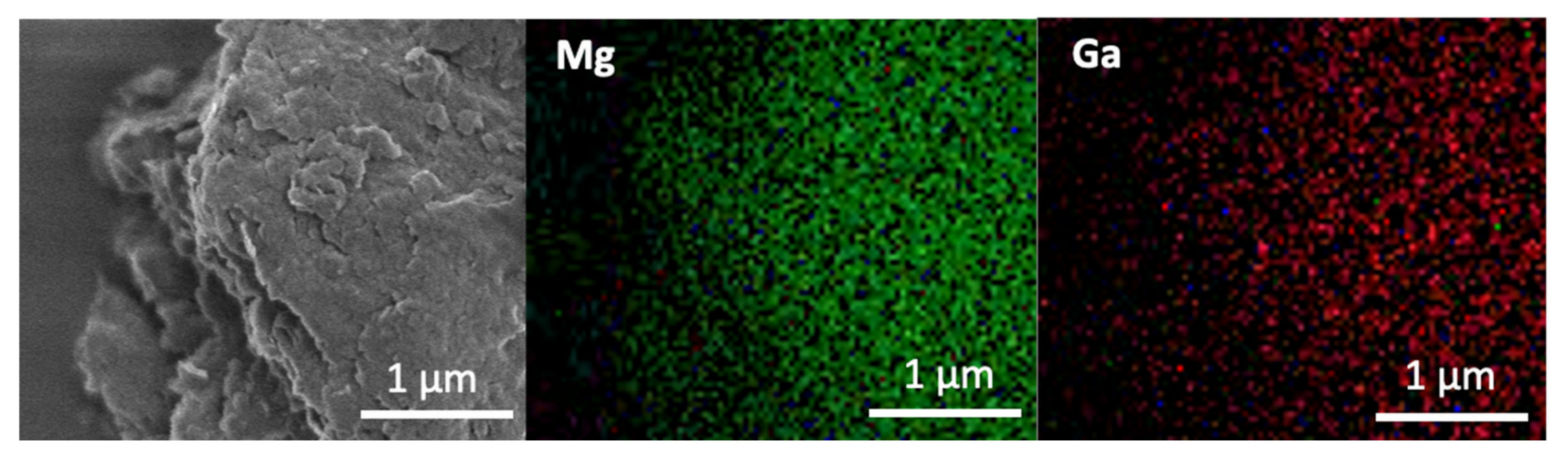
SEM image and elemental mapping of MgGa(I)-0.06. The element was presented by Mg (green) and Ga (red).

The chemical composition of the products, which was determined by ICP analysis, is summarized in Table 1. The x values (M^3+^/(M^2+^ + M^3+^) ratios) of the products prepared at the pH of the mixture at around 11–12 were smaller than those in the starting solution. It was reported that the loss of Ga as soluble Ga complexes in the preparation of MgGa–LDH at the pH above 10 [28]. In addition to the chemical composition determined by ICP analysis, the distribution of Mg and Ga in the platy particles was examined by EDS elemental mapping (Figure 4) equipped with SEM, showing the uniform distribution of Mg and Ga through the products. The Ga/I ratios in the products were larger than 1.0, suggesting the possible coexistence of some anions with I^-^ to compensate the positive charge by the MgGa substitution. As the coexisting interlayer anion, the presence of nitrate anion in the products was confirmed by the absorption due to NO stretching vibration at 1342 cm^−1^ (MgGa(I)-0.24) and 1350 cm^−1^ (MgGa(I)-0.11 and MgGa(I)-0.06) observed in the IR spectra (Figure 3).

The zeta potential of the products is summarized in Table 1. The trend of the values correlated with the composition and the values are different from that of the commercial brucite, supporting the possible variation of layer charge density, which depends on the composition.

**Figure 5:**
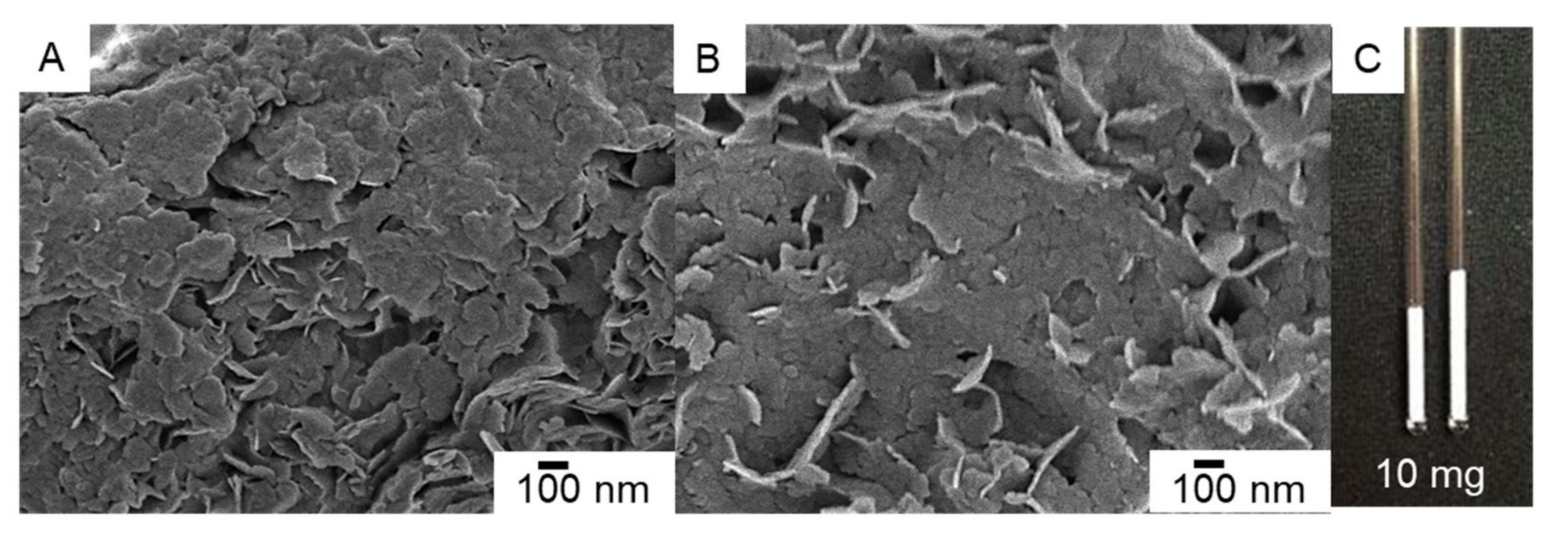
SEM images of MgGa(I)-0.06 (**A**) and MgGa(I)-0.06_F (**B**). (**C**) Photograph of the products in a capillary tube to show the difference in the density, 10 mg of sample in the capillary without compressing; MgGa(I)-0.06 (left) and MgGa(I)-0.06_F (right).

The area per charge (and the layer charge density) of MgGa(I)-0.24, MgGa(I)-0.11 and MgGa(I)-0.06 were estimated from the x value and the lattice parameter *a* of the products as summarized in Table 1. Due to the lower layer charge density, MgGa(I)-0.06 was expected to be dispersed in water better if compared with MgGa(I)-0.24. In order to accelerate the dispersion, the samples were freeze dried to obtain LDH particles with less aggregation for stable dispersion. The samples dried by heating and by freeze drying showed a difference in the particle aggregation, while the freeze-dried sample had a lower degree of aggregation. The difference derived from the drying method was seen by the SEM images of MgGa(I)-0.06 and MgGa(I)-0.06_F (Figure 5A,B). This difference is reflected to the apparent density as shown by the photograph of the samples in the capillary tube (Figure 5C), where 10 mg of MgGa(I)-0.06_F had larger volume in the capillary than MgGa(I)-0.06 (corresponding to lower bulk density). Note that all samples exhibited platy particles in their SEM images to support the successful formation of LDHs.

**Figure 6:**
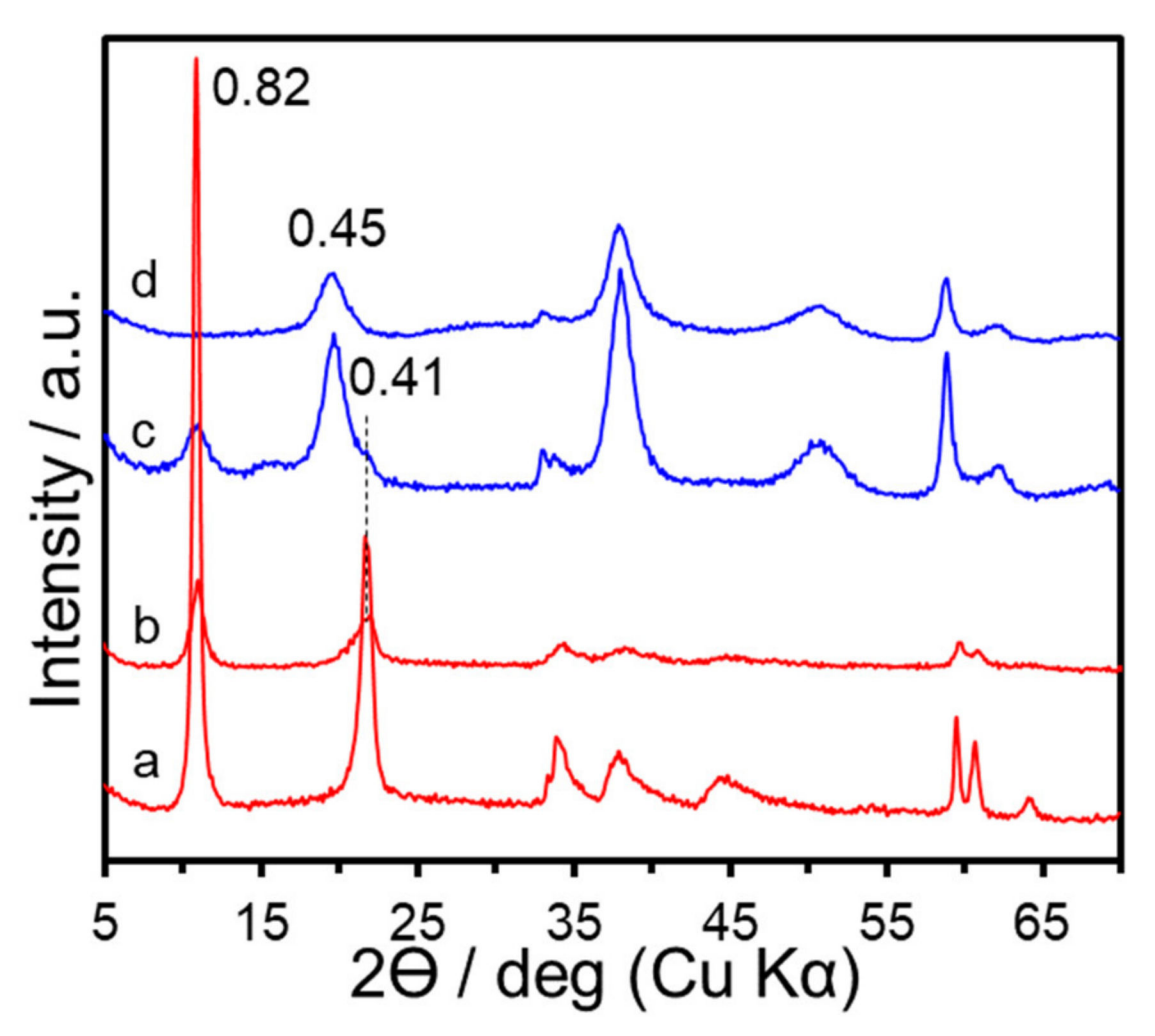
XRD patterns of MgGa(I)-0.24 (**a**), MgGa(I)-0.24_F (**b**), MgGa(I)-0.06 (**c**), and MgGa(I)-0.06_F (**d**).

The XRD patterns of the freeze-dried samples (MgGa(I)-x_F, x = 0.24 and 0.06) are shown in Figure 6 together with the corresponding samples dried by heating (MgGa(I)-x, x = 0.24 and 0.06). For the products with x of 0.24, the *(003)* and *(006)* reflections with the d values of 0.82 and 0.41 nm were observed in both MgGa(I)-0.24 and MgGa(I)-0.24_F (Figure 6a,b), suggesting the regular stacking of iodide intercalation in the interlayer of the MgGa–LDH. On the contrary, for the products with x of 0.06, the XRD pattern of MgGa(I)-0.06 (Figure 6c) showed the characteristic reflections of interstratified structure with the basal spacings of 0.82 and 0.45 nm as schematically shown in Figure 2. The *(003)* reflection was not seen for MgGa(I)-0.06_F (Figure 6d), suggesting an irregular layer stacking. This difference between MgGa(I)-0.24_F (regular stacking) and MgGa(I)-0.06_F (irregular stacking) was thought to be caused by the difference in the swelling due to the difference in the layer charge density, which is expected to correspond to the x value.

**Figure 7:**
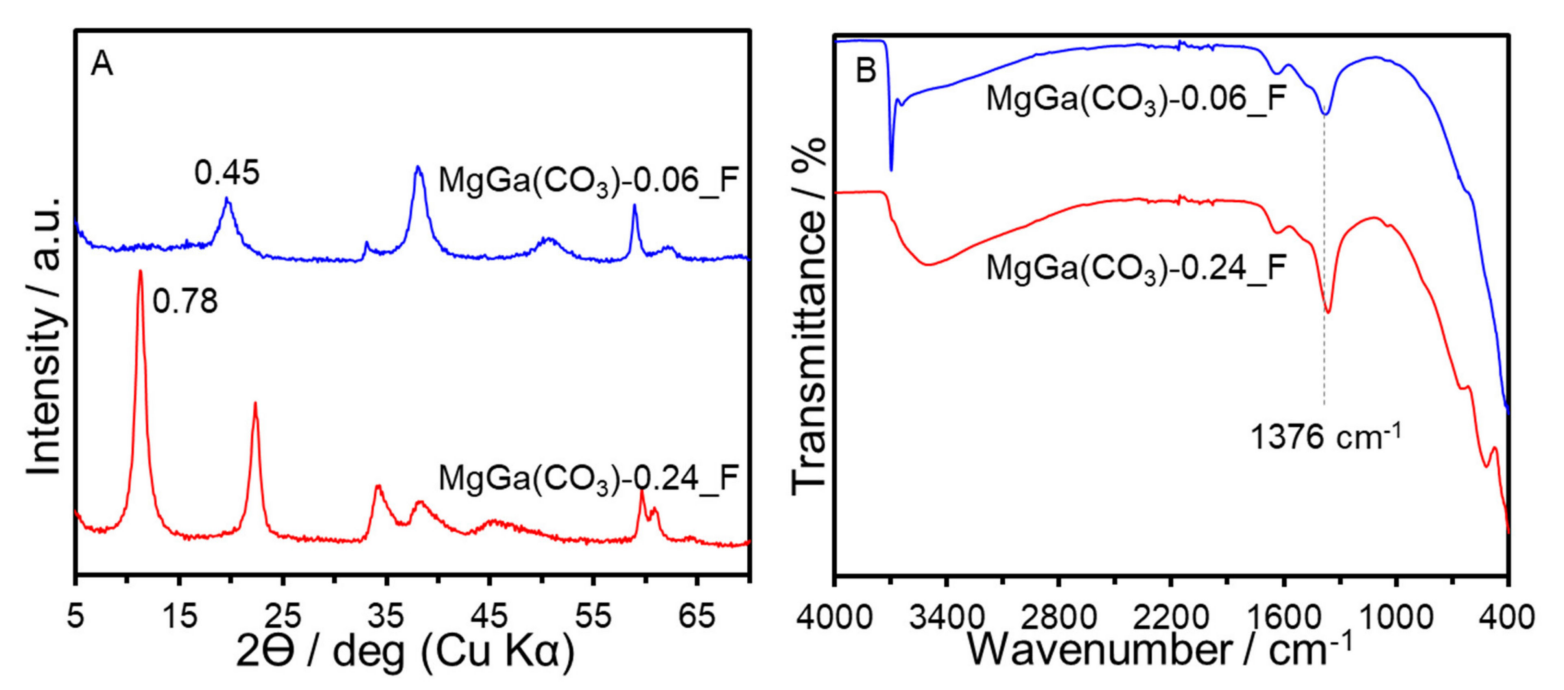
(**A**) XRD patterns and (**B**) IR spectra of MgGa(CO_3_)-0.24_F and MgGa(CO_3_)-0.06_F.

The carbonate forms of LDH were prepared by the ion exchange with sodium carbonate. The ion exchange of the interlayer anion of LDHs results in the change in the interlayer spacing. To induce the ion exchange, the electrostatic interactions between LDH layer and exchanging anions should be larger than that between LDH layer and original anions [12,36]. The XRD patterns and IR spectra of MgGa(CO_3_)-0.24_F and MgGa(CO_3_)-0.06_F are shown in Figure 7A,B. MgGa(CO_3_)-0.24_F showed the basal spacing of 0.78 nm, which is consistent with the basal spacing of MgGa–LDHs containing carbonate anion as the interlayer anion [11], indicating the successful ion exchange of iodide and nitrate anions with carbonate. The results are in agreement with the reported sequence of the ion exchange of MgAl–LDH as the order of iodide < nitrate < carbonate [28]. The basal spacing of MgGa(CO_3_)-0.24_F (0.78 nm) was smaller than MgGa(I)-0.24_F (0.82 nm), which is related to the size of anions as CO_3_^2−^ = 0.38 nm and I^-^ = 0.42 nm [37,38,39]. For MgGa(CO_3_)-0.06_F, the *(003)* reflection was not observed in the XRD pattern as shown in Figure 7A, suggesting the irregular layer stacking. It was thought that the MgGa(CO_3_)-0.06_F swollen to some extent during the anion exchange thanks to the lower layer charge density, resulting in the irregular stacking by applying freeze drying. The anion exchange was indicated by the IR spectrum (Figure 7B), where the absorption due to the C-O stretching vibration of carbonate anion in the interlayer was seen at 1376 cm^−1^. The layered structure was maintained after the ion exchange of the iodide with carbonate. This suggested that the collapsing of the layered structure in the previous studies [32,33] occurred during the formation (precipitation) of LDH and once layered structure formed, the structure was relatively stable to accommodate different anions by the ion exchange.

**Figure 8:**
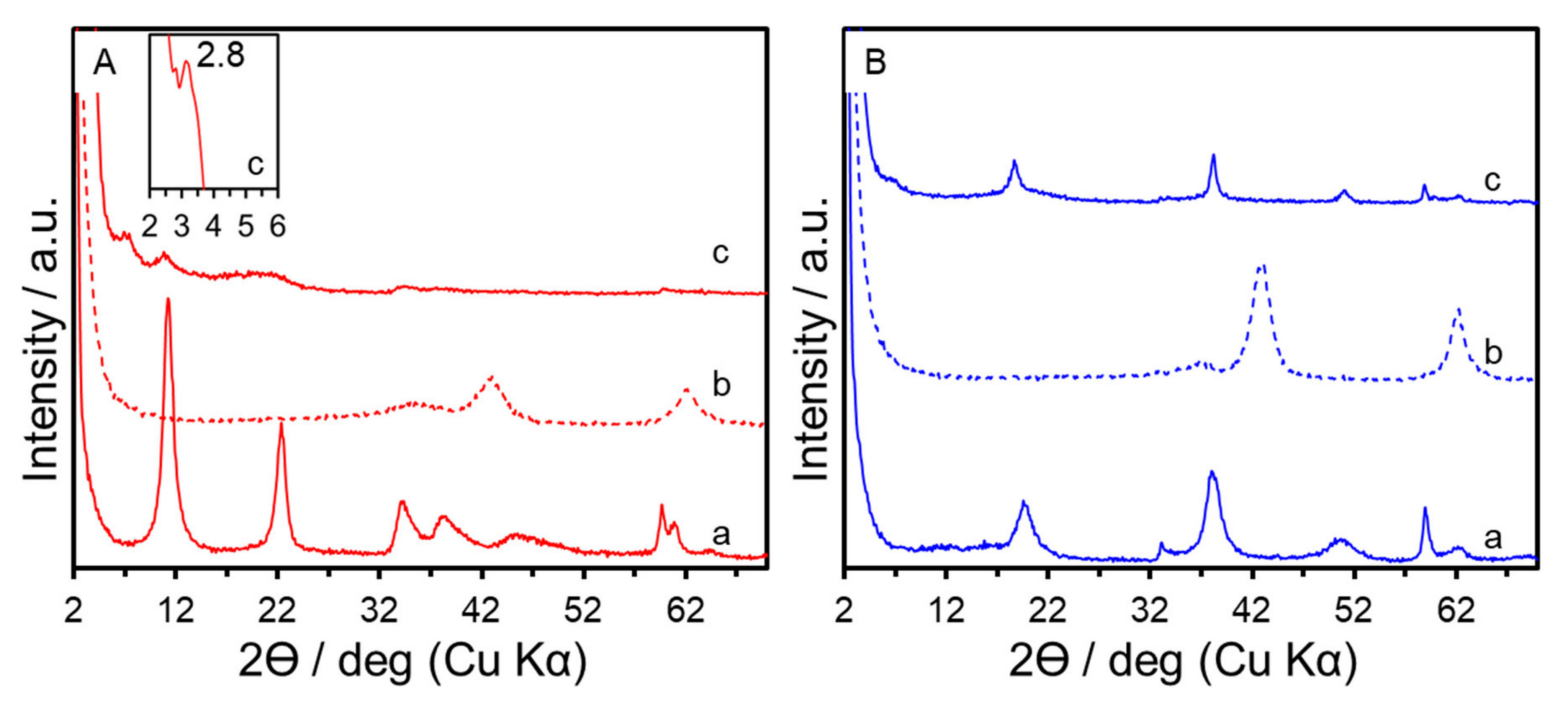
XRD patterns of MgGa(CO_3_)-x_F (a), calcined MgGa(CO_3_)-x_F (b), and MgGa(DS)-x_F (c); x of 0.24 (**A**) and 0.06 (**B**). Inset: the *(003)* reflection of MgGa(DS)-0.24_F at 2*θ of 3*°.

**Figure 9:**
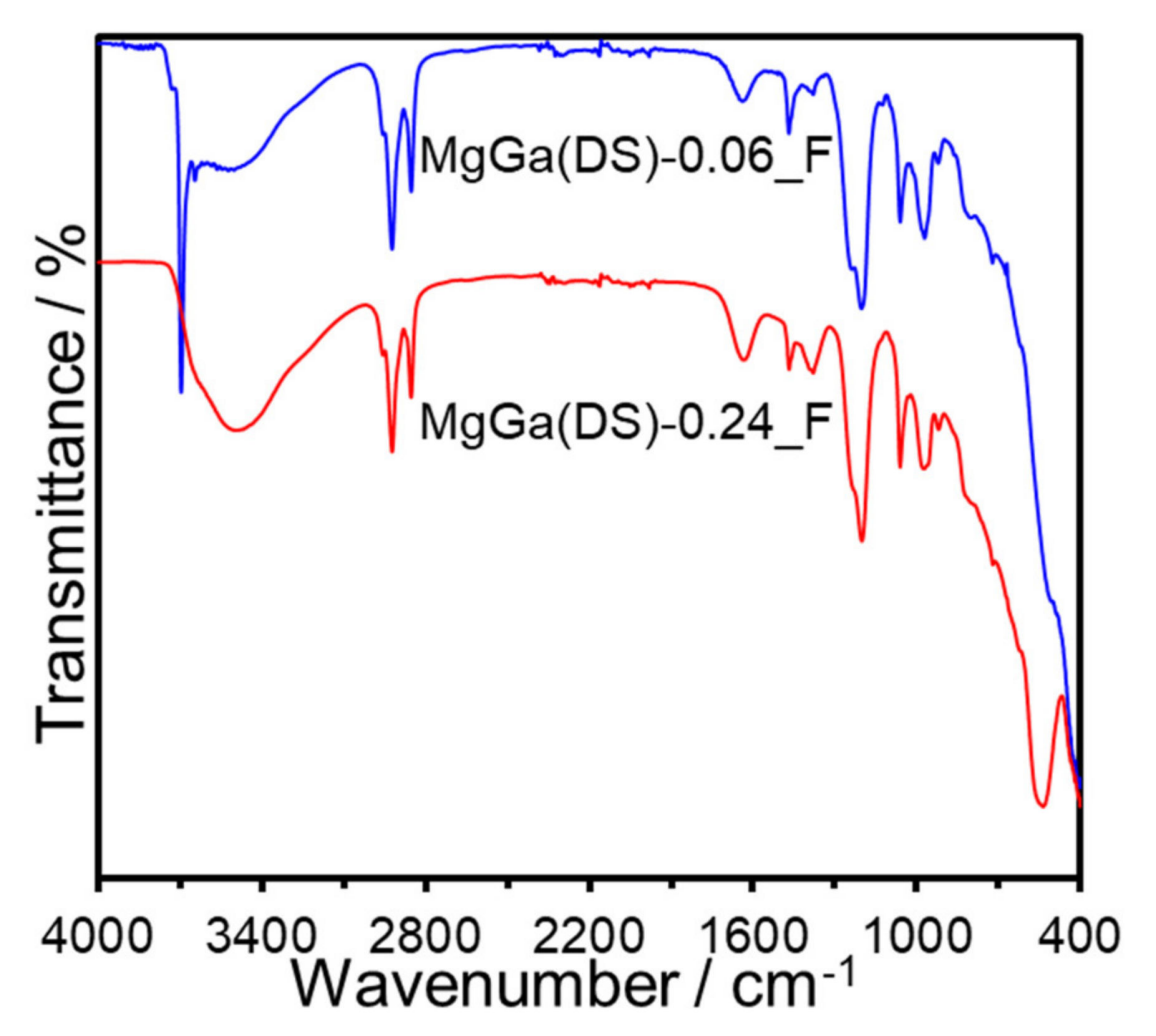
IR spectra of MgGa(DS)-0.24_F and MgGa(DS)-0.06_F.

Reconstruction of MgGa(CO_3_)-0.24_F and MgGa(CO_3_)-0.06_F were examined by the calcination at 500 °C and subsequent reaction with the aqueous solution of sodium dodecyl sulfate (SDS). The calcination of MgGa(CO_3_)-x_F (x = 0.24 and 0.06) at 500 °C caused the dehydration, decarbonation, and dehydroxylation to give mixed metal oxides, which is known to form LDH structure by up-taking the anion from aqueous solution [16]. The transformation to the oxide by the calcination was confirmed by XRD patterns (Figure 8A(b),B(b)), where diffraction peaks at *2*θ of 36, 42, and 62° (corresponding to the characteristic reflections of periclase (MgO) phase (JCPDS No. 04-0829)) were observed. The oxide reconstructed to the original LDH structures after the reaction with SDS as shown by the XRD patterns (Figure 8A(c),B(c)). The XRD pattern of MgGa(DS)-0.24_F exhibited the basal spacing of 2.8 nm (Figure 8A (inset)), suggesting the incorporation of dodecyl sulfate (DS) anion in the interlayer space. If compared with the reported MgAl with x of 0.32 [40], the basal spacing of MgGa(DS)-0.24_F was smaller because of the difference in the layer charge density of the host. The layer charge density was reported to influence the orientation of DS in the interlayer space [41,42,43]. For MgGa(DS)-0.06_F, the *(003)* reflection was not seen in Figure 8B(c), suggesting the irregular stacking, which was thought to be caused by the swelling due to the lower layer charge density. The incorporation of DS anion in MgGa(DS)-0.06_F was confirmed by the absorption at 2922 and 2853 cm^−1^ due to C-H stretching vibration and those at 1200 and 1058 cm^−1^ due to S=O stretching vibration observed in the IR spectrum of MgGa(DS)-0.06_F (Figure 9). The IR results are consistent with those for the reported MgAl LDH containing DS as the interlayer anion [44].

**Figure 10:**
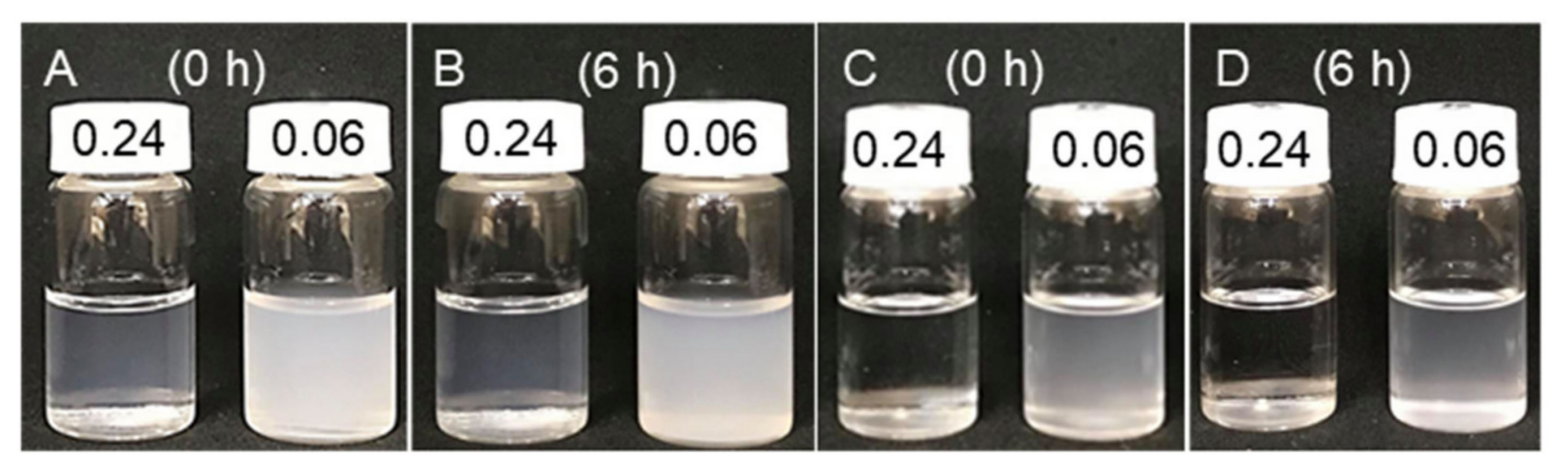
Photographs of dispersion of MgGa(CO_3_)-x_F in water (**A**,**B**) and MgGa(DS)-x_F in hexanol (**C**,**D**) before and after the storage for 6 h.

The effect of x value on the dispersion was examined using MgGa(CO_3_)-x_F (x = 0.24 and 0.06) to be dispersed in water. Figure 10A,B shows the photograph of the dispersion before and after the storage for 6 h without agitation. MgGa(CO_3_)-0.24_F sedimented while MgGa(CO_3_)-0.06_F was dispersed. The dispersion of MgGa(DS)-x_F (x = 0.24 and 0.06) in hexanol was also examined and the appearance of the dispersion are shown in Figure 10C,D. MgGa(DS)-0.06_F was dispersed in hexanol, while MgGa(DS)-0.24_F sedimented. Since particles sizes were not different significantly for the samples, the difference in the dispersion behavior was thought to be a result of the difference in the layer charge density.

## 4. Conclusions

MgGa–LDHs with varied x of 0.06–0.24 were prepared by coprecipitation using iodide as interlayer anion. The linear correlation between lattice parameter *a* and the x values as well as the uniform distribution of Mg and Ga through the particles supported the successful substitution of Mg in the brucite-like sheet with Ga at x of 0.06–0.24. Interstratification was suggested for the sample with smaller x values, while those with larger values of 0.18 < x < 0.24 showed regular stacking of LDH layers with intercalated anion in c-axis. The zeta potential supported the possible variation of layer charge density due to the difference in the x values. Once layered structures of the MgGa–LDHs with varied x formed, the MgGa–LDHs with x of 0.06 and 0.24 were exchanged with carbonate by ion exchange and reconstructed with dodecyl sulfate after calcination, which were confirmed by the change in the basal spacing and the existence of stretching vibrations of the anions in the interlayer space. For the effect of x value on the dispersion, the carbonate type MgGa–LDH with x of 0.06 was dispersed in water and the MgGa–LDH with x of 0.06 containing dodecyl sulfate was dispersed in hexanol better than those of MgGa–LDHs with x of 0.24, suggesting possible effects of the layer charge density on the dispersion.

## Figures and Tables

**Table 1:** Summary of the chemical composition, the estimated area per charge, the estimated layer charge density, and zeta potential of the products and the commercial brucite.

Sample	x _(starting solution)_	x _(products)_	Ga/I _(products)_	Estimated Area per Charge (nm^2^/(+)) ^1^	Estimated Layer Charge Density ((+)/nm^2^) ^2^	ζ-Potential (mV)
MgGa(I)-0.24	0.25	0.24	2.0	0.35	2.86	38.71
MgGa(I)-0.18	0.20	0.18	2.1	0.47	2.13	NA.^3^
MgGa(I)-0.11	0.13	0.11	2.4	0.76	1.31	34.47
MgGa(I)-0.10	0.10	0.10	2.7	0.93	1.07	NA.^3^
MgGa(I)-0.08	0.09	0.08	2.9	1.07	0.93	NA.^3^
MgGa(I)-0.06	0.08	0.06	3.4	1.31	0.76	30.81
Mg(OH)_2_	-	-	-	-	-	16.94

^1^ Estimated area contains one positive charge = a^2^sin60^o^/x [35]. ^2^ Layer charge density = 1/(estimated area per charge). ^3^ NA. = not analyzed.

## Data Availability

The data presented in this study are available on request from the corresponding author.
